# On a Case of Progressive Muscular Atrophy: Demonstrating the Nervous Origin and Nature of the Disease, and Its Amenability to Nervine Tonics

**Published:** 1887-06

**Authors:** Charles Williams

**Affiliations:** District Medical Officer of the Bodmin Union, Admiralty Surgeon, &c.


### Clinical Records.

---

### ON A CASE OF PROGRESSIVE MUSCULAR ATROPHY: DEMONSTRATING THE NERVOUS ORIGIN AND NATURE OF THIS DISEASE, AND ITS AMENABILITY TO NERVINE TONICS.

By CHARLES WILLIAMS, L.R.C.P., L.R.C.S., L.S.A., District Medical Officer of the Bodmin Union, Admiralty Surgeon, &c.

In the month of April, 1883, J. B. M., by profession a mariner, and aged 48 years, came to me complaining of weakness in the left arm and pain in the fingers and thumb of the same side. As for the wrist, he told me me that it "felt exactly as if it were broken, and the fractured ends were grating one upon the other;" adding at the same time that he had "no strength in it all."

On examination of the affected limb, I found the interossei muscles and the thenar and hypothenar eminences unmistakably wasted, and also the same mischief—only more marked still—in the muscles of the forearm and the biceps. So greatly wasted, indeed, were these latter-named muscles, that, compared with their fellows on the other side, they felt quite soft and flabby. Moreover, whilst with the sound limb closure of the hand and flexion of the forearm on the arm would produce a feeling of firmness and resiliency, a similar movement with the affected limb only served to demonstrate more palpably the serious damage its muscles had sustained.

Of course, the case was so clear that I could hardly make a mistake in diagnosis; but still, the malady being one not often met with in country practice, I deemed it wise, before definitely pronouncing an opinion, to investigate its history. If any doubt existed before, it was now speedily dispelled; for when to the characteristic symptoms he presented was added such a pathognomonic history as he now briefly, but afterwards so fully and graphically, detailed to me, how could I possibly remain any longer in uncertainty?

On my telling him what I feared his malady was, the serious nature of it, and how improbable the prospect of a complete recovery, he replied that he was not at all surprised, that he had himself feared it was something serious, for it had been coming on for such a long time; "in fact," said he, "ever since I was shipwrecked that time." And then, to my request for a detailed history of the whole case from the beginning, he furnished me with the following interesting narrative: "I am, as you know, a sea-captain, and in 1875, when I was out in rough weather in Bude Bay, my vessel collided with the rocks, and was completely wrecked. All the crew were almost at once taken off in the rocket apparatus; but, unfortunately, just as my turn came the apparatus broke down. What my exact feelings were at this juncture it would be impossible for me to describe. Suffice it to say that I was completely terror-stricken, lights seemed to flash before my eyes, and I gave up all hope of being saved. Once, I remember a wave struck me with such tremendous force, and threw me so violently on my back, that those on shore told me afterwards that they thought it was all over with me. I

regained my equilibrium, however, and for two hours, clothed in nothing but my drawers, made frantic endeavours to get into the apparatus. Ultimately, by a terrific jump—I hardly know now how I did it—I succeeded in getting one leg in. Directly those on shore saw that I had reached it they began to pull, and the consequence was that I was dragged almost the whole way under water. Just as I was nearing the land, the sea was so violent that it knocked me completely off the breeches* into the water; and had not the sailors jumped in and secured me that very instant, I should certainly have been drowned. From this moment I completely lost my senses, and the next thing I remember is finding myself in bed at a gentleman's house, whither I had been carried. At the end of three days I was brought home here in a closed conveyance. Of course the doctor was sent for immediately, and he continued to attend me for several months. I was as long as six weeks in bed, even; and during that time had most peculiar delusions. For instance, everything appeared to me as if topsy-turvy: my wife would look like a hand-spike, and several times I distinctly remember I fancied the house was going head over heels. These hallucinations, I may say, would not be constant, but would pass and come. In addition to all this, I remember, too, that I used to have dreadful pain in my head; and this, so far as I can recollect, was almost constant. About the treatment, I know that I had for many months to take strengthening medicine, and several times I remember I had injections.†

"After that I got fairly well, but still not myself by any

* The "breeches" is the term employed to designate that part of the apparatus in which the legs of a shipwrecked person are wont to be placed.

† It was evidently morphia that was injected; for it was done to relieve his intense pain, and he always slept, so he said, afterwards.

means. For instance, I was very nervous—could never trust myself, and so forth—and, besides this, I know I had, too, jerking pains in my arm. Owing to my being so very nervous, I remained at home for three or four years, and then only went to sea because I was obliged to. After seven or eight months, however, finding it wouldn't do, I came home again, and this time determined never to attempt it more. Whilst at home this second time I used to earn my livelihood by going out with the other men fishing; my most prominent symptoms the while being such as I have just stated.*

"I continued in this state, getting no better and becoming no worse; when one day, when I was out fishing, the boats were caught in a 'ground-sea,'† and the lives of myself and the other fishermen were for a time in great peril. Fortunately, however, the lifeboat was speedily got out, and we were all ultimately rescued. Although we escaped with nothing worse, apparently, than a severe wetting, yet this shock affected me, I know, not a little, for afterwards I got more nervous than ever.

"Such was my condition, with very little difference, till the spring of 1882, when news reached the village that the vessel which I had been to sea in last had foundered on the coast of Scotland. It was managed by two nephews of mine; and as from the report all hands seemed undoubtedly to be lost, I, as a near relative and a seafaring man besides, was deputed by the friends, in the absence of anyone else apparently so suitable, to proceed to the spot and try to identify and bring home the bodies of these two young men. I felt at the time that it was not at all the thing for me, and the result proved that my fears

* That is, nervousness, want of confidence, &c.

† More correctly, "ground-swell."

were well grounded." He then proceeded to give me the details of his mournful errand: how that, sad at heart and hardly knowing what he was doing, he started that afternoon from this out-of-the-way fishing village, and, after a tedious journey, extending from the afternoon of Tuesday to midnight on the following Thursday, he found himself in the town of Dundee. Arrived here, he discovered to his chagrin that a mistake had been made as to the locality, and that the wreck had occurred nowhere near here, but in the neighbourhood of the town of Aberdeen. Proceeding thither with as little delay as possible, he found himself a few hours later at the scene of the disaster. When the man came to this part of his narrative he was visibly affected, and when he had told me what he had to go through I was not surprised at it. Amongst other painful experiences, there was the recognition of the vessel with which he had so long been familiar as going gaily before the winds, now a complete wreck; and embedded in the sands. Then there was a visit to a mortuary, where, laid out on slabs, were the bodies of about a dozen drowned sailors, and over whom, just as he entered, the burial service was in solemn tones being read. The impression all this was likely to make upon him may be imagined. Now, however, came the worst part of all; for each face of these twelve men—cold and grim in death, and decomposed in some cases beyond any possible recognition—he had, so soon as the service was concluded, to carefully and deliberately scan. When to this is then added the fact that he had to come away from that "palace of the dead" without even the sad satisfaction of feeling that *his* dead were amongst them, some idea will have been gained of the terrible trial to which his nerves must have been subjected. Other painful episodes also occurred, which I have here no space to mention; but the end of it all was, that he had to make his long return journey without the object of his mission having been accomplished. The results to himself, then, of this nerve-trial were, if my theory of his malady be correct, exactly as might have been expected; for, from that time forward his symptoms grew steadily, if not rapidly, worse, and, after a couple of years of slow but sure retrogression, he presented himself a patient at my surgery. What state he was in at that time has been already described, and his condition whilst under my care and inspection I will allude to presently. Here, for a moment, let me draw attention to the first point I want to lay stress upon in this article, and that is: that progressive muscular atrophy—the malady we are considering—has its origin, not, as some have supposed, in a disturbance of the muscular system, but undoubtedly and unmistakably in a disturbance of the nervous. I know, of course, that the primary lesion is now generally admitted to be of this nature; but still, for all that, even at the time of writing, I also know equally well that there are not a few who regard the whole thing, from beginning to end, much in the same light as they do the degeneration of a fatty heart. That I am quite correct in my assertion is evident from the fact that even to-day that designation of Duchenne's, "Atrophie musculaire avec transformation grasseuse," and other equally misleading appellations, still pass current amongst us. This erroneous nomenclature, however, there is no doubt is gradually becoming obsolete, and, as I have just intimated, the nervous hypothesis is the now generally accepted explanation of the matter. But, if this be so, how is it that more stress is not laid in medical text-books upon nervous shock as an exciting cause of the disease?

*A priori*, one would certainly expect this would be one of the most common of all. And yet in the only medical works to which I have had access—and one of them is one of the latest and most esteemed medical works that the last decade has produced—the occurrence is not so much as hinted at. Dr. Roberts—for this is the authority to whom I am alluding—in the sixth edition of his work on *The Theory and Practice of Medicine*, thus sums up the pathology and ætiology of this disease: "Wasting palsy (that is, progressive muscular atrophy) has been attributed pathologically to an atrophic and degenerative change beginning in the involved muscles themselves, in the anterior roots of the nerves supplying them, or in the spinal cord. The view which now seems to be generally held, is that the primary lesion consists in a gradual destruction of the motor nerve-cells of the anterior cornua of the cord, which some regard as due to a chronic inflammation, others to a degeneration, the muscles being secondarily affected. The chief supposed exciting causes are, exposure to cold and wet; a blow or fall on the neck or back, or traumatic injuries of peripheral parts; syphilis; excessive use, with consequent fatigue, of the affected muscles; acute fevers; and lead-poisoning." Now this work is admitted on all hands to be one of the best modern expositions of present-day medicine; and yet, with all modesty, may I be permitted to suggest that it would not detract in the least from the value of the work if in a future edition there was added after the first paragraph of the quotation given above, "*or in the brain;*" and after the last paragraph, "*nervous shock*"?

My justification for such a suggestion is to be found in the case I am now placing upon record; for, whatever the cause and wherever the mischief in other cases, in *this* I

am persuaded they are none other than intimated above. That is to say, whilst "nervous shock" was undoubtedly, as it seems to me, the exciting cause of the disease, "in the brain" would, I believe, be found the primary lesion. And upon anyone who has closely followed the history of this man's malady, so graphically related by him, the same conviction must, I think, be impressed. If, however, the mere recital of its commencement (in this connection note the hallucinations, &c., which were months anterior to anything noticeably wrong with the muscles) were not sufficient to convince, I would draw attention to the fresh impetus given the malady by his trying trip to Scotland; and also, only in a lesser degree, by the fright he suffered when the fishing boats were imperilled. Quite recently, too, he has suffered another relapse, equally suggestive; but this I will allude to again.*

Here let me leave this the first part of my subject, and proceed to the second. What, then, were the course and character of his symptoms whilst a patient under my care? Well, under the treatment adopted (the precise nature of this will be detailed further on; here I will only mention that it was both constitutional and topical) the man unmistakably improved. Whilst the muscles of the affected limb gradually regained a good deal of their original firmness, the improvement showed itself still further by the increased strength he got in it. Unfortunately, however, coincidently with this improvement in the left arm the malady attacked the right leg. Under the application of the same local treatment however, and a continuance with the medicine, after a time the leg improved also. When the leg was attacked, both the man and myself feared that

* *Vide* note on page 111.

the disease was going to invade the whole body, and our conversations on the subject went so far as to lead us to discuss the precise mode in which he would probably die. Our apprehensions, however, did not continue long; for, as the disease showed no signs of attacking the other leg and arm and the muscles of the trunk, it soon became evident to both patient and doctor that they could fairly congratulate themselves on the enemy having been routed.* With matters in this state, then, the local treatment was discontinued, and he did nothing but persevere with the medicine. After another twelve months, or about two years from the time of his first presenting himself at my surgery, he discontinued this too (at all events, its *regular* use), and, with a few slight relapses,† he has remained in much the same state ever since.

Such, then, is a brief *resumé* of the course of the malady locally. Now let me proceed to give more in detail a few of his general symptoms, or what should more correctly, perhaps, be called his "sensations." What, then, were these? Well, as will be seen, they were very peculiar. Strange to say, too, they seemed—or, at all events, some of them did—to be largely dependent upon the condition of the atmosphere. Thunder weather, for instance, used to affect him very strikingly.

* The disease was not cured, but arrested; in fact, the man is not *cured* even now.

† His last relapse was, however, rather a severe one. He was a candidate for the post of Attendance Officer under our local School Board. A member of that board myself, and having some influence with it, I went so far as to tell him that he could calculate upon obtaining the appointment. The result of a vote by ballot, however, showed that a rival had secured it. I told his wife at once that her husband would now be sure to have another relapse; and, surely enough, although the man by my direction made up his mind that he would not allow his disappointment to affect him, in a week or two afterwards my prognostications were verified.

Often and often he used to close the shutters and go to bed; and nearly always when this kind of weather was coming on he would come to, or send for, me. At such times, too, —but occasionally at other times as well—he would experience a sensation as if the house were going over, and the invariable result would be that he would fall down on his knees and catch hold of something. At other times, again,—and the symptoms about to be narrated would be apparently independent of atmospheric influences—his sensations would be "as if a live animal were in his shoulder-joint and gnawing away at the tissues." Again and again he has fancied—and *apropos* of this I am satisfied that this sensation was just like the others, namely, nervous,—that "whole armies of lice" were moving about his shoulder; and several times a day —once he assured me he did it as often as sixteen—he has stripped himself completely naked to find out whether it really were so or not. Almost similar sensations, too, he has had in the affected thigh. Thus, he would come complaining that the feeling was exactly as if a horsewhip had struck it, and he felt the tingling that follows; or sometimes it would be not this, but a feeling like a lot of spiders playing over it. Sometimes, again, the sensations would be painful. Thus, he would feel pain akin to what he fancied would be experienced if the bones were bending; or the feeling of spiders would be succeeded by a sharp twinge, just as if a sharp knife were suddenly plunged in; or, if it was not this, it was just as if a pistol-barrel had been applied to his leg and the bullet was passing through it. Then, again, in addition to his remarkable sensations, there were peculiar involuntary movements. Involuntary twitchings of the muscle, as might be guessed, were, of course, of almost continuous occurrence, but occasionally they would be much more than this. Thus, sometimes when he would be sitting in a chair, without any warning, one leg would jump involuntarily over the other knee. I say "involuntarily," because he assured me that it was so, and because on one occasion I saw the phenomenon myself, and I know that I was not deceived.

Thus, I think it will have to be admitted that both his symptoms and sensations were peculiar in the extreme. So peculiar were they, indeed, and so numerous (for I have not related, I know, one half of them), that I fancy some of my readers will shake their heads when they read about them. Assuming this to be the case, let me anticipate all the objections that it seems to me can possibly arise out of a perusal of what has been recounted. If it be said that the man was not telling the truth, let me assure my readers that I know otherwise. I could always see it in his face and manner; and not only so, but what object could he have in deceiving me? If it is argued that some of his experiences were indicative of an over-indulgence in alcohol, my reply is that, for what he drank, he might almost be called a teetotaller. If it be urged that the man was, perhaps, failing mentally, my answer is that, on the other hand, he was most intelligent; and, beyond what I have related here, his actions were in no way eccentric. Then, lastly, if it be suggested that some of the symptoms were probably due, more or less, to the treatment adopted, I will only say that, whilst the local treatment was soon discontinued, the medicine that I commenced with was never changed; that when he was taking it, he always improved; and that when he was not, he always got worse. Thus all objections, so far as I can see, being anticipated and disposed of, what is the lesson to be learnt from his case ? None other, I take it, than this: that the disease in its origin and nature is essentially and unmistakably nervous. But, if I am correct and the disease be indeed nervous, then nervine treatment ought to effect an improvement. Well, so it did. Recognising the fact that the disease clearly showed absence of tone in the nervous system, it was not difficult to see that nervine tonics and not nervine sedatives were what were required. What, then, to bring the matter to a point, was the treatment adopted ? Well, as has been mentioned, it was both internal and external. The internal consisted in administering a mixture of steel and strychnine, and the external in the local application to the affected muscles of the magneto-electric current. The medicine was taken uninterruptedly for as long as two years, while the local treatment was discontinued after about twelve months.* As to the part each played in the resulting improvement my own opinion is very decided, and that is, that while both combined helped to give the first check to the disease, the medicine alone has held it back since. Asked what ingredient in this mixture I attribute most virtue to, I should unhesitatingly answer that it was the strychnine. And yet, strange to say, this drug is dismissed as worthless in Dr. Tanner's discussion of this malady. He says, speaking of his treatment of this disease: "It may be instructive to mention *(sic)* that amongst the remedies which have certainly failed to do good we must place strychnine and nux vomica, setons, issues, or blisters over the vertebræ, and cold baths during the active stage." † He is probably right about the setons, &c., but

* Whilst it lasted, however, it was done almost daily, either by myself, my pupil, or both of us.

† *Vide* Dr. Tanner's *Practice of Medicine*, 7th edition, vol. 1, p. 477.

he is certainly in error about the strychnine. At all events, if he was not *then*, he is *now*; for this case proved, as clearly as the value of any medicine could be proved, the benefit derived from strychnine. Whilst in the beginning he persevered with it regularly, and under it slowly but surely made progress; afterwards, when better, he always used to come whenever there seemed to be a slight relapse, and a bottle or two of the mixture almost invariably pulled him together again.* Met by the suggestion that it was probably the steel as much as the strychnine that worked such good results, I have only to say that I have strong presumptive evidence to the contrary. It so happened that a few months ago he had one of his slight relapses, and came to me as usual for his "pick-me-up." Of course I gave it him, and when he had finished taking it he came again for a second, saying that he was much better, and he thought another bottle would put him all right. Now, it chanced that when he came the second time my stock of liquor strychnine was all but exhausted, and so I was only able to put in his bottle just half the quantity he always had had before. Of course I never thought anything more of it, but about a week afterwards he again presented himself at my surgery, and, almost immediately after he had entered the room, he made use of these (to me) very significant words: "I don't know how it is, but that last bottle did not do me so much good as the other." Without a doubt, then, we have in this drug, and probably in other nervine tonics as well, a remedy of proved value; and if any of my professional brethren should be confronted with a similar case, I trust, after what has been said, that it will

* The precise composition of the mixture was liq. ferri. perchlor. m. x to a dose, and liq. strychnine m. v. to a dose.

not be necessary for me to urge them to give it or its kindred drugs a fair trial.

And here I take leave of this interesting case, feeling sure that by placing it on record, I have established beyond doubt—first, that this malady is nervous in its origin; secondly, that it is nervous in its nature; and, thirdly and lastly, that, in at least some cases, it will be found amenable to nervine tonics.

